# Modified Hybrid Repair with Open Reconstruction of Infrarenal Aortoiliac Segment for Thoracoabdominal Aortic Aneurysms Without Custom Distal Landing Zone: A Single-Center Case Series

**DOI:** 10.3390/jcm15218542

**Published:** 2026-11-02

**Authors:** Pengchao Duan, Linyao Zhu, Jiarong Wang, Luyu Chen, Ding Yuan, Tiehao Wang, Jichun Zhao, Bin Huang

**Affiliations:** 1 Division of Vascular Surgery, Department of General Surgery, West China Hospital, Sichuan University, Chengdu 610041, China; 2 West China School of Medicine, Sichuan University, Chengdu 610041, China; 3 Department of General Surgery 1, West China Tianfu Hospital, Sichuan University, Chengdu 610213, China

**Keywords:** thoracoabdominal aortic aneurysm, modified hybrid repair, multibranched prosthetic graft, stent-graft exclusion, vascular surgery, case series

## Abstract

**Background/Objectives:** Inadequate distal landing zones can limit hybrid repair of thoracoabdominal aortic aneurysm (TAAA). We evaluated a modified strategy combining prosthetic distal landing-zone reconstruction, retrograde visceral and renal revascularization, and thoracic stent-graft exclusion. **Methods:** This retrospective single-center case series included all nine consecutive patients with inadequate native distal landing zones who underwent single-stage modified hybrid repair in 2025. Open aneurysm excision and aortoiliac reconstruction provided the distal sealing segment and inflow for visceral and renal bypasses. The primary outcome was 30-day all-cause mortality. Secondary outcomes included postoperative length of hospital stay, 30-day major complication rate, 30-day paraplegia rate, 1-year reintervention rate, 30-day readmission rate, and technical success rate. **Results:** Five patients had Crawford extent II aneurysms and four had extent III aneurysms. Technical success was achieved in all nine patients. Median operative time, estimated blood loss, and intensive care unit stay were 724 min, 2500 mL, and 8 days, respectively. Thirty-day all-cause mortality was 0% (0/9). No spinal cord ischemia or mesenteric ischemia occurred within 30 days. Acute kidney injury occurred in six patients (66.7%), including one who received continuous renal replacement therapy. One patient required early covered stent-graft repair of a right iliac anastomotic leak. At a median follow-up of 9 months (IQR, 6–12), all reconstructed visceral and renal bypasses were patent, no new endoleak was detected, and all patients were alive. No additional reintervention was required. **Conclusions:** Modified hybrid repair with prosthetic distal landing-zone reconstruction was technically feasible in selected TAAA patients with inadequate distal sealing anatomy. Larger studies with longer follow-up are needed to evaluate durability and refine indications.

## 1. Introduction

Thoracoabdominal aortic aneurysm (TAAA) represents one of the most challenging conditions in aortic surgery because successful repair requires exclusion of extensive aneurysmal disease while maintaining adequate perfusion of the spinal cord, visceral arteries, and renal arteries. The selection of an appropriate treatment strategy depends on multiple factors, including aneurysm extent, patient characteristics, anatomical feasibility, and the ability to achieve durable proximal and distal sealing [1,2,3].

Open repair remains an established treatment option for selected patients, particularly those with connective tissue disorders or unfavorable endovascular anatomy [3,4]. However, it requires extensive surgical exposure, aortic reconstruction, and complex perioperative management [5]. Fenestrated or branched endovascular repair has expanded the treatment options for high-risk patients but remains dependent on suitable landing zones, target-vessel anatomy, device availability, and institutional expertise [6,7]. In patients with aneurysmal involvement of the distal abdominal aorta or iliac arteries, an adequate distal landing zone may not be available, which can limit the feasibility of endovascular exclusion [1,8].

Hybrid repair combines open visceral debranching with endovascular exclusion and has been adopted as an alternative strategy for selected patients with complex TAAA anatomy [9]. By avoiding extensive thoracic aortic replacement, hybrid approaches may reduce procedural invasiveness in appropriately selected patients [10,11]. However, conventional hybrid repair generally relies on the native distal aorta or iliac arteries as the distal sealing segment for subsequent stent-graft deployment [12]. When these segments are affected by aneurysmal degeneration, an insufficient distal landing zone may remain a major technical limitation despite successful visceral artery debranching.

To address this anatomical limitation, our center developed a modified hybrid strategy that combines prosthetic reconstruction of the distal aortic segment with retrograde visceral and renal artery debranching. During the open phase, the diseased distal abdominal aorta and/or iliac arteries are reconstructed using a prosthetic graft, which subsequently serves as the distal landing zone for thoracic stent-graft exclusion. This approach allows simultaneous restoration of visceral and renal perfusion while creating a suitable sealing segment for endovascular exclusion of the residual thoracoabdominal aneurysmal segment.

This study reports our initial experience with this modified hybrid strategy in nine consecutive patients with TAAA and inadequate distal landing zones. We evaluated patient selection, anatomical characteristics, operative details, early clinical outcomes, and short- to mid-term imaging findings. This case series is reported in accordance with the PROCESS guideline [13,14].

## 2. Materials and Methods

### 2.1. Study Design

This retrospective, single-center case series was conducted at our center. All consecutive patients with thoracoabdominal aortic aneurysms (TAAAs) who underwent modified hybrid repair between January and December 2025 were retrospectively reviewed.

The primary objective of this study was to assess 30-day all-cause mortality following modified hybrid repair in patients with TAAA and inadequate distal landing zones. Secondary objectives were to assess postoperative length of hospital stay, 30-day major complications, 30-day paraplegia, reintervention within 1 year, 30-day readmission, and technical success.

### 2.2. Patient Selection

Patients were considered candidates for modified hybrid repair when conventional hybrid repair was considered unsuitable because of inadequate distal sealing zones for thoracic endovascular aortic repair (TEVAR).

The primary anatomical indication was aneurysmal involvement of the distal abdominal aorta and/or iliac arteries, resulting in the absence of an adequate native distal segment for durable stent-graft fixation.

All patients underwent preoperative computed tomography angiography (CTA) with three-dimensional reconstruction. A multidisciplinary team reviewed aneurysm extent, visceral artery anatomy, iliac artery condition, and the feasibility of open reconstruction and subsequent stent-graft exclusion.

Preoperative CTA was used to assess stenosis or occlusion of the native celiac artery, superior mesenteric artery, and bilateral renal arteries. For descriptive reporting, stenosis severity was categorized using a study-specific five-category classification: grade 0, no stenosis; grade 1, mild stenosis (>0% to <50%); grade 2, moderate stenosis (50–70%); grade 3, severe stenosis (>70% to <100%); and grade 4, complete occlusion [15,16]. The mild, moderate, and severe categories followed published angiographic conventions, whereas the numerical grades were assigned for descriptive reporting in this study.

Patients were included if they met all of the following criteria: (a) diagnosis of TAAA requiring surgical treatment; (b) absence of an appropriate distal landing zone for endovascular exclusion because of distal abdominal aortic or iliac artery involvement; (c) the presence of an infrarenal abdominal aortic segment with a short-axis diameter of less than 40 mm.

Patients undergoing conventional hybrid repair without distal landing-zone reconstruction, isolated abdominal aortic repair, staged procedures, or other hybrid strategies were excluded.

### 2.3. Definition of Modified Hybrid Repair

Modified hybrid repair comprised open prosthetic reconstruction of the diseased abdominal aortic segment to establish inflow, retrograde revascularization of the indicated visceral and renal target vessels, and thoracic stent-graft exclusion of the residual proximal aneurysm.

The modified hybrid strategy was designed to create a prosthetic distal landing zone when the native distal aorta or iliac arteries could not provide an adequate sealing segment for the planned stent-graft repair.

**Figure 1:**
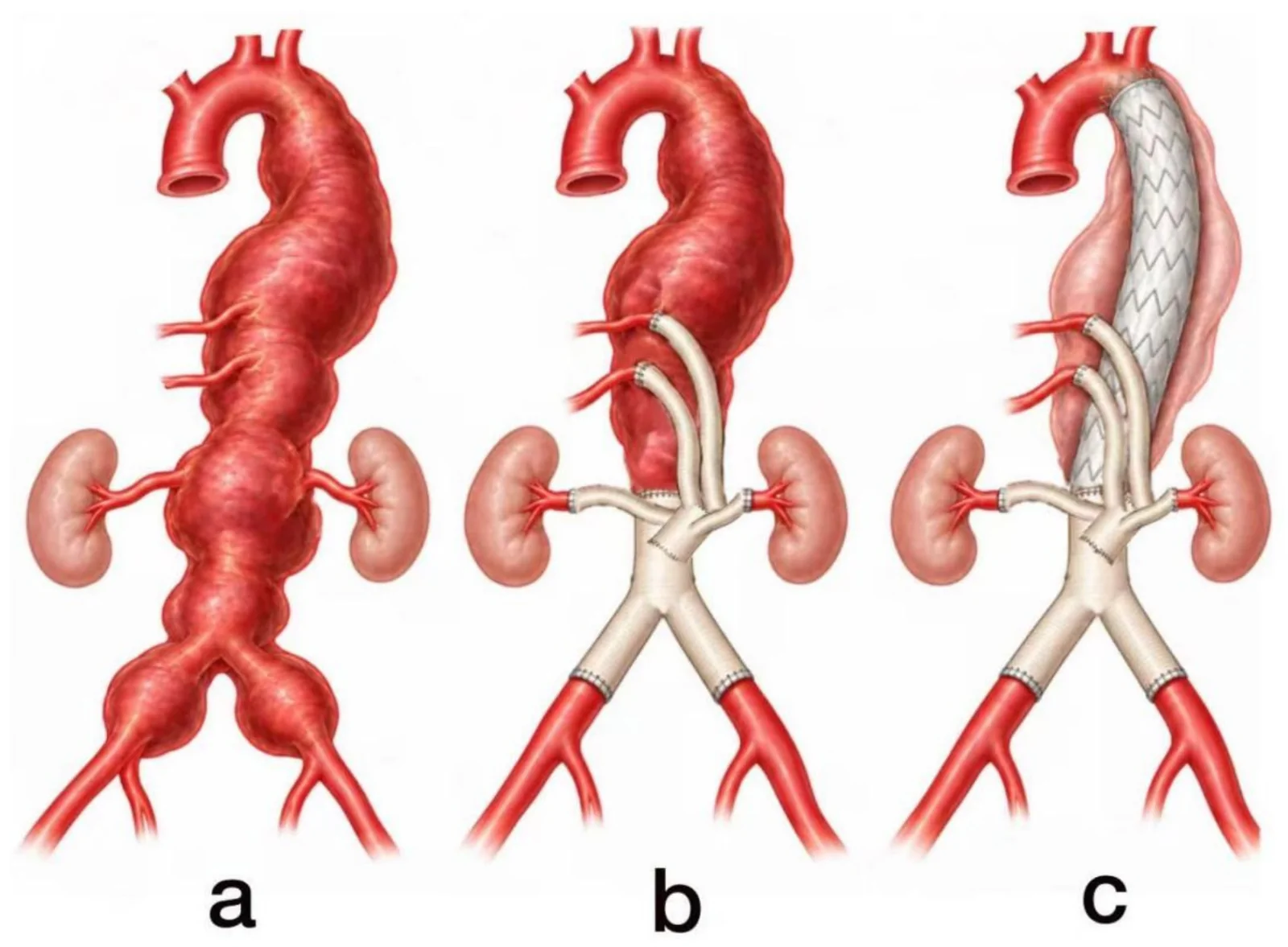
Modified hybrid repair for thoracoabdominal aortic aneurysm. (**a**) Preoperative thoracoabdominal aortic aneurysm involving the descending thoracic aorta, suprarenal abdominal aorta, abdominal aorta, and visceral branch segment. (**b**) Open reconstruction after abdominal aneurysm excision. The abdominal aorta and iliac inflow are replaced with a multibranched prosthetic graft, and retrograde bypass branches are anastomosed to the celiac trunk, superior mesenteric artery, and bilateral renal arteries. (**c**) Final repair after proximal stent-graft exclusion. A thoracic covered stent graft excludes the residual suprarenal or thoracoabdominal aneurysmal segment, while the multibranched prosthetic graft maintains visceral and renal artery perfusion.

During the open phase, the affected infrarenal abdominal aorta was replaced with a prosthetic graft, with iliac reconstruction performed as required. The proximal portion of the reconstructed aortic graft provided the distal sealing segment for subsequent thoracic stent-graft deployment, while the graft also served as inflow for retrograde visceral and renal artery revascularization (Figure 1b). A thoracic stent graft was then used to exclude the residual suprarenal or thoracoabdominal aortic disease (Figure 1c). Anatomical eligibility for this reconstruction strategy required the presence of an infrarenal abdominal aortic segment with a short-axis diameter of less than 40 mm.

### 2.4. Operative Technique

All procedures were performed by experienced vascular surgeons as single-stage repairs in a hybrid operating room under general anesthesia. A midline transperitoneal incision exposed the abdominal aorta, iliac arteries, and planned visceral and renal target vessels. The celiac trunk was approached through the lesser sac, and the proximal superior mesenteric artery was exposed cranial to the renal arteries.

**Figure 2:**
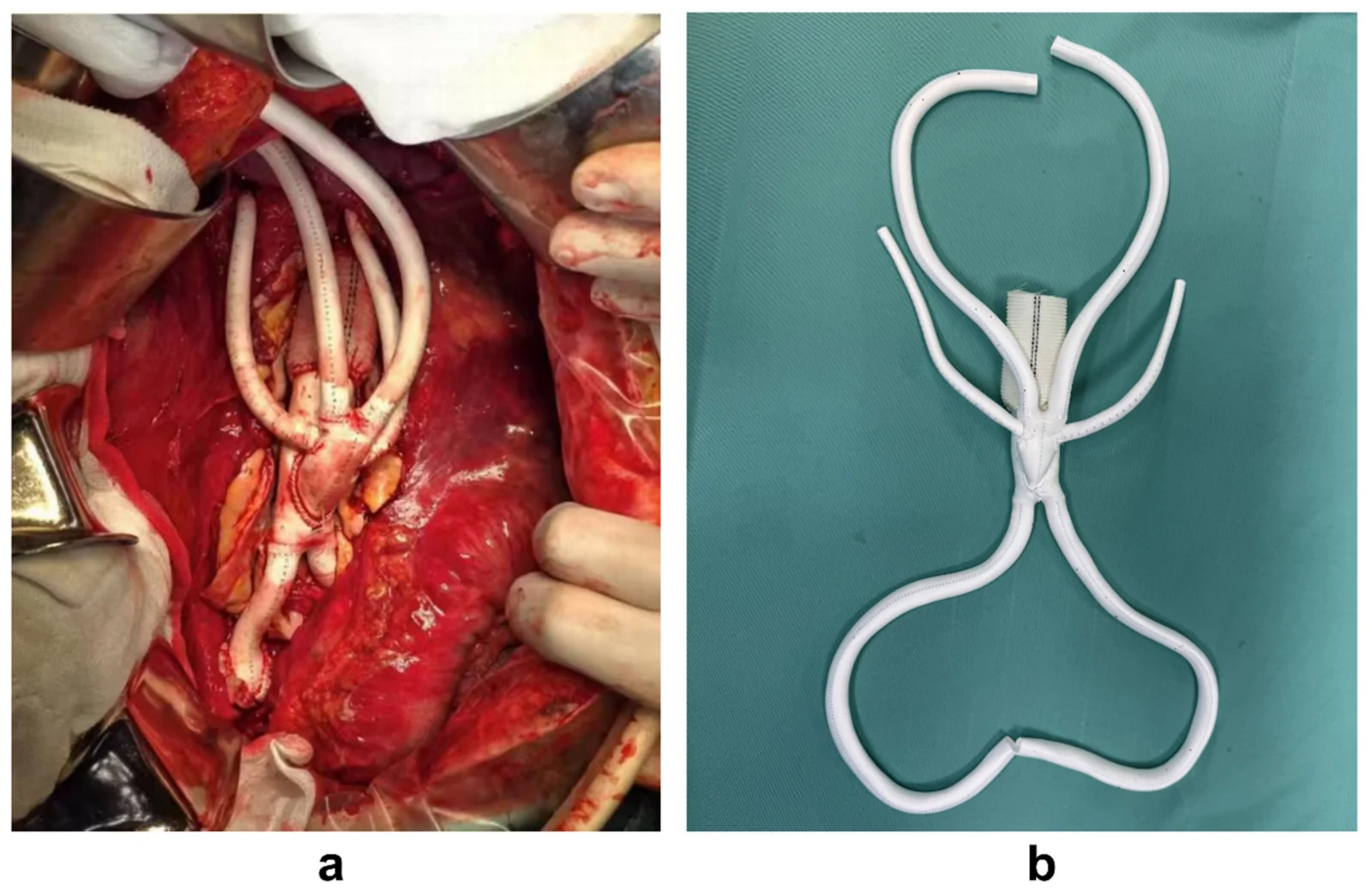
Intraoperative photographs of the open reconstruction. (**a**) Intraoperative photograph after completion of the open reconstructive phase, showing the implanted prosthetic graft and retrograde visceral and renal bypass branches. (**b**) Composite prosthetic graft configuration. A 24-mm straight double-velour woven graft was connected to an 18 × 9-mm bifurcated graft for aortoiliac reconstruction, with an inverted multibranched graft sewn onto the graft body to enable retrograde visceral and renal artery revascularization.

Two graft constructs were prepared during vessel exposure. The aortoiliac conduit comprised a 24-mm straight double-velour woven graft (MAQUET Cardiovascular LLC, Wayne, NJ, USA) sewn to the proximal end of an 18 × 9-mm bifurcated GORE-TEX vascular graft (W. L. Gore & Associates, Inc., Flagstaff, AZ, USA). A separate four-branch debranching conduit was fashioned by suturing two additional 6-to-4-mm tapered GORE-TEX grafts to openings in a 16 × 8-mm bifurcated GORE-TEX graft, with branch dimensions selected according to target-vessel diameter (Figure 2a,b). CV-3 or CV-5 GORE-TEX sutures were used for graft assembly and inflow anastomoses.

The infrarenal aorta was cross-clamped and the aneurysmal segment was excised. The 24-mm woven segment was anastomosed to the infrarenal aorta, and the distal bifurcated limbs were anastomosed to the iliac arteries at levels determined by disease extent. The reconstructed graft was then clamped, and an opening was created in its bifurcated body. The four-branch conduit was inverted, with its branches directed cranially, and its main trunk was anastomosed end-to-side to this opening.

The branches were routed retrogradely to the celiac circulation, superior mesenteric artery, and bilateral renal arteries (Figure 1b). Renal reconstruction preferentially used end-to-end anastomoses, whereas the superior mesenteric artery was reconstructed end-to-side near its origin. The celiac bypass was passed beneath the pancreas and anastomosed end-to-side at the celiac bifurcation or splenic artery, according to exposure and anatomy. Target-vessel anastomoses used PROLENE polypropylene sutures with HEMO-SEAL technology (Ethicon, Inc., Somerville, NJ, USA): 6-0 for celiac and superior mesenteric reconstruction and 7-0 for renal reconstruction. The native proximal origins of all reconstructed visceral and renal arteries were securely suture-ligated to prevent retrograde perfusion of the excluded aneurysmal segment (Figure 2a). Warm ischemia time was defined as the interval from target-vessel clamping to final unclamping and restoration of blood flow through the completed bypass. This interval was recorded separately for each visceral and renal target vessel. 

After open reconstruction, the bypass inflow location was marked for identification during the endovascular phase. Percutaneous common femoral access was established using two predeployed Perclose ProGlide closure devices (Abbott Vascular, Santa Clara, CA, USA), followed by a 16F sheath (Cook Medical, Bloomington, IN, USA) for angiography. Angiography confirmed bypass patency and defined the planned stent-graft coverage. Contrast medium was diluted 1:1 with normal saline. The proximal stent-graft end was deployed within a previous descending thoracic stent graft or the native descending thoracic aorta distal to the left subclavian artery origin. The distal end was positioned within its proximal prosthetic segment, below the infrarenal anastomosis and above the bypass inflow opening.

Completion angiography assessed aneurysm exclusion, proximal and distal sealing, reconstructed vessel patency, and the absence of type I or III endoleak. Hemostasis was secured, the retroperitoneum was closed, and no abdominal surgical drain was placed.

### 2.5. Perioperative Management

Preoperative assessment included cardiopulmonary and renal function, nutritional status, and coagulation status. Prophylactic cerebrospinal fluid drainage was placed before surgery in all nine patients. Warming blankets were used to maintain normothermia and support coagulation function during the prolonged open and endovascular phases.

Invasive arterial pressure monitoring guided volume resuscitation and vasoactive support. Intraoperative management targeted a mean arterial pressure of 90–100 mmHg when feasible to support spinal cord, renal, and mesenteric perfusion. Cell salvage was used, and allogeneic blood products were administered when required. Reduced-dose systemic heparinization was guided by the activated clotting time (ACT), with a target of approximately 1.5 times the baseline value. ACT was rechecked hourly, and additional heparin was given when needed to maintain the target. No adjunctive renal perfusion or cooling technique was used; renal protection relied on limiting renal artery interruption during reconstruction, maintaining circulating volume, and avoiding sustained hypotension.

All patients were admitted to the intensive care unit after surgery. Monitoring included blood pressure, lower-limb and neurological examinations, abdominal findings, urine output, serum creatinine, acid-base status, lactate, and respiratory function. Prophylactic low-molecular-weight heparin was administered postoperatively, with timing and dose adjusted according to renal function, coagulation status, and bleeding risk. Subsequent supportive care was individualized according to postoperative recovery.

### 2.6. Outcomes and Follow-Up

The primary outcome was 30-day all-cause mortality, defined as death from any cause within 30 days after the index procedure, regardless of whether it occurred during hospitalization or after discharge. Secondary outcomes were postoperative length of hospital stay, 30-day major complication rate, 30-day paraplegia rate, 1-year reintervention rate, 30-day readmission rate, and technical success rate. Bypass patency, endoleak, and survival at the last follow-up were recorded as additional descriptive outcomes.

Major postoperative complications were defined as Clavien–Dindo grade III or higher events occurring within 30 days after the index procedure [17]. The major complication rate was calculated as the proportion of patients experiencing at least one such event among all nine patients. Each patient was counted once, and the highest complication grade was used for patient-level severity reporting.

Postdischarge reintervention was defined as any additional open or endovascular procedure related to the treated aorta or reconstructed grafts within 12 months after discharge from the index hospitalization. Reinterventions performed during the index hospitalization were reported separately. Unplanned readmission was defined as an unplanned hospital admission within 30 days after discharge from the index hospitalization. Technical success was defined as completion of the planned open abdominal aortic or iliac reconstruction, intended visceral and renal revascularization, and thoracic stent-graft exclusion. Completion angiography was required to demonstrate satisfactory stent-graft position and sealing, patent reconstructed inflow and intended bypasses, and no type I or type III endoleak.

Postoperative acute kidney injury (AKI) was retrospectively assessed using the Kidney Disease: Improving Global Outcomes (KDIGO) 2012 criteria: an increase in serum creatinine of ≥26.5 μmol/L within 48 h, an increase to ≥1.5 times baseline within 7 days, or urine output <0.5 mL/kg/h for at least 6 h [18,19]. Preoperative serum creatinine was used as the baseline. Serum creatinine and urine-output criteria were evaluated according to the availability and temporal resolution of the original records, and the highest ascertainable stage was reported.

Spinal cord ischemia was defined as new lower-limb motor or sensory dysfunction attributed to spinal cord hypoperfusion. Mesenteric ischemia was diagnosed from clinical findings, laboratory results, imaging, and surgical exploration when required.

Follow-up was scheduled at 1, 6, and 12 months after surgery and annually thereafter. Assessments included clinical examination, renal function testing, and CTA. Imaging surveillance focused on stent-graft position and sealing, endoleak, residual aortic disease, anastomotic abnormalities, visceral and renal bypass patency, and indications for reintervention.

Because the abdominal aneurysm was excised during the open phase, abdominal aneurysm sac regression was not assessed. Follow-up imaging instead evaluated the residual proximal aortic segment and both the open and endovascular components of the repair. The date of each patient’s last clinical follow-up coincided with the date of the last follow-up CTA.

### 2.7. Statistical Analysis

Descriptive statistics were used. Continuous variables are reported as mean ± standard deviation or median with interquartile range, as appropriate. Categorical variables are reported as counts and percentages. No comparative, regression, or survival analyses were performed because of the small sample size and absence of a control group. Statistical analyses were conducted using R version 4.4.3.

## 3. Results

### 3.1. Patient Characteristics

All nine patients who underwent modified hybrid repair at our center in 2025 were included in the analysis. Seven were men (77.8%), and the median age was 43 years (IQR, 36.5–70). The median left ventricular ejection fraction was 63% (IQR, 59–68), the median estimated glomerular filtration rate was 108.0 mL/min/1.73 m^2^ (IQR, 93.8–111.4), and the median B-type natriuretic peptide level was 95 pg/mL (IQR, 37–165).

The most common comorbidities were chronic pulmonary disease in 6 patients (66.7%) and hypertension in 5 (55.6%). Marfan syndrome and peripheral artery disease were each present in 2 patients (22.2%), and diabetes mellitus was present in 1 (11.1%). Four patients (44.4%) had dissecting thoracoabdominal aortic aneurysms, and five (55.6%) had nondissecting thoracoabdominal aortic aneurysms. Five patients (55.6%) had Crawford extent II aneurysms and 4 (44.4%) had extent III aneurysms. Baseline characteristics are summarized in Table 1.

### 3.2. Anatomical and Landing-Zone Characteristics

**Figure 3:**
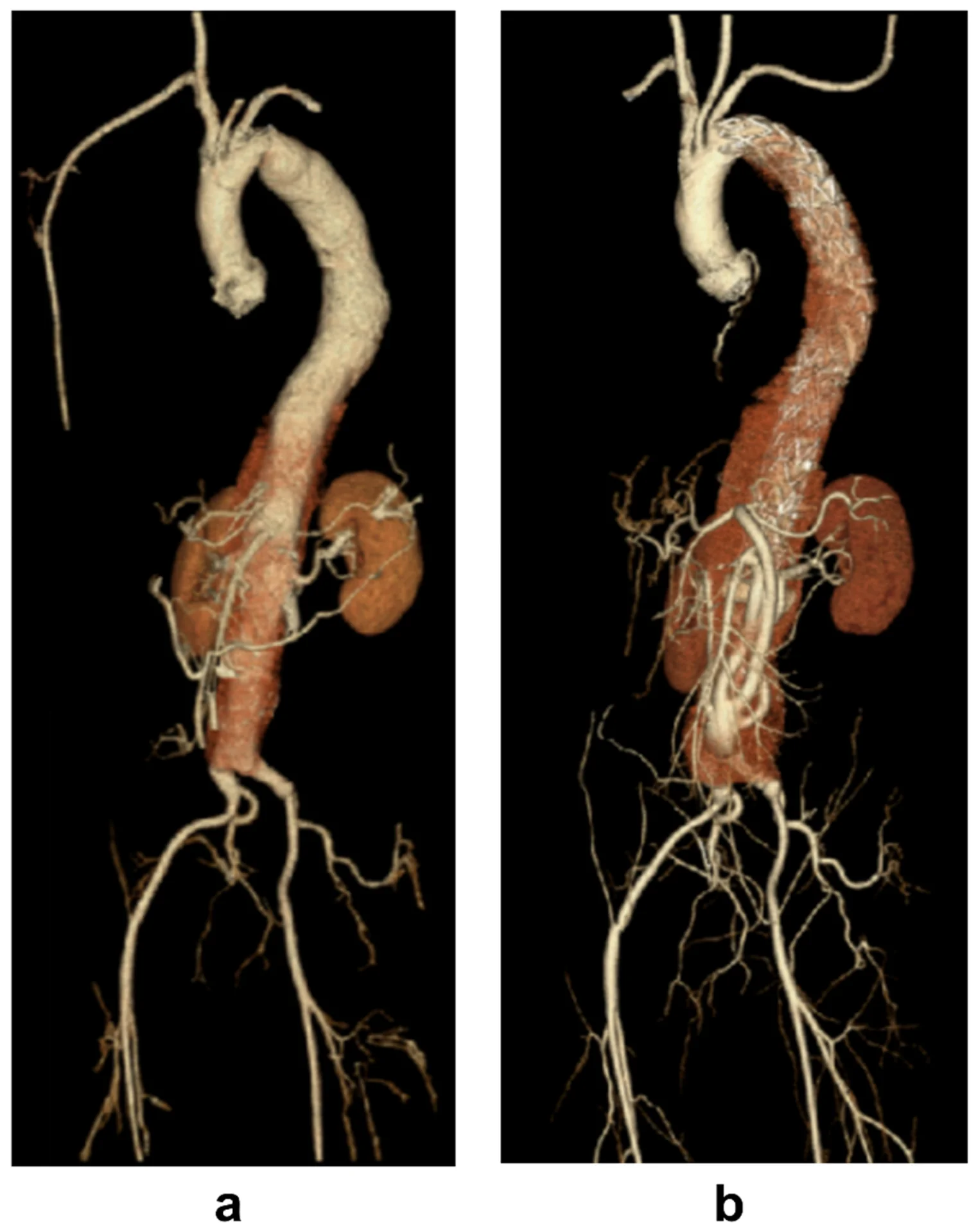
Preoperative and postoperative three-dimensional computed tomography angiography reconstructions. (**a**) Preoperative reconstruction showing the thoracoabdominal aortic aneurysm. (**b**) Postoperative reconstruction showing the thoracic covered stent graft, the reconstructed abdominal aortic and iliac segment, and the visible retrograde visceral and renal bypasses.

All patients had inadequate native distal landing zones due to aneurysmal involvement of the distal abdominal aorta and/or iliac arteries. The median maximum aneurysm diameter was 58.0 mm (IQR, 57.0–64.5). Iliac artery aneurysms were present in two patients (22.2%). No patient had left subclavian artery involvement or severe bilateral iliac artery stenosis or occlusion. Right iliac artery tortuosity with an angulation of 30–60° was present in two patients (22.2%). A representative preoperative three-dimensional CTA reconstruction is shown in Figure 3a.

Target-vessel patency was generally preserved. The superior mesenteric artery and left renal artery were free of stenosis in all patients. The celiac trunk was classified as grade 0 in 6 patients (66.7%), grade 1 in 2 (22.2%), and grade 2 in 1 (11.1%). The right renal artery was classified as grade 0 in 7 patients (77.8%), grade 1 in 1 (11.1%), and grade 2 in 1 (11.1%).

Four patients (44.4%) had previously undergone descending thoracic aortic stent-graft placement. In these patients, the proximal landing zone was located within the existing stent graft and had a median diameter of 27.5 mm (IQR, 24.8–28.0). In the remaining 5 patients, the proximal landing zone was located in the native descending thoracic aorta distal to the left subclavian artery origin. The median native landing-zone diameter was 28.0 mm (IQR, 23.5–31.0). Anatomical characteristics are summarized in Table 2.

### 3.3. Procedural Details

All nine patients underwent single-stage modified hybrid repair. The superior mesenteric artery and bilateral renal arteries were reconstructed in all nine patients. The celiac trunk was reconstructed in eight patients, and the splenic artery was used as an alternative target in one patient because the celiac trunk could not be adequately exposed. Median warm ischemia times were 19.0 min (IQR, 16.5–21.5) for the celiac artery, 18.0 min (IQR, 12.0–20.0) for the superior mesenteric artery, 23.5 min (IQR, 20.5–29.0) for the left renal artery, and 19.0 min (IQR, 17.5–27.5) for the right renal artery. The warm ischemia time for the single splenic artery reconstruction was 25.0 min (Table 2). Bilateral common iliac artery reconstruction was performed in seven patients and unilateral right common iliac artery reconstruction in two. One patient also underwent left external iliac artery reconstruction. The visceral and renal bypass inflow was supplied by an infrarenal prosthetic graft. In patients with a previous descending thoracic stent graft, the new proximal device was extended into the existing graft. In the remaining patients, the proximal device was deployed in the native descending thoracic aorta distal to the left subclavian artery origin. The distal landing zone was selected according to the extent and configuration of the abdominal prosthetic reconstruction. No intraoperative deviations from the final preoperative plans were recorded in the nine cases.

### 3.4. Early Outcomes

Thirty-day all-cause mortality was 0% (0/9). The median operative time was 724 min (IQR, 657.5–742), and the median estimated blood loss was 2500 mL (IQR, 1400–5500). The median blood transfusion volume was 1150 mL (IQR, 375–3650), and the median autologous blood transfusion volume was 1350 mL (IQR, 975–2125). The median intensive care unit stay was 8 days (IQR, 5–9).

Postoperative AKI occurred in six patients (66.7%) according to the KDIGO criteria. The highest documented stage was stage 1 in three patients, stage 2 in two, and stage 3 in one. One patient received continuous renal replacement therapy (CRRT) in the setting of persistent postoperative creatinine elevation and progressively increasing B-type natriuretic peptide levels, with suspected heart failure with preserved ejection fraction.

The renal complication requiring CRRT was classified as Clavien–Dindo grade IVa. One patient (11.1%) developed a right iliac anastomotic leak within 30 days and underwent covered stent-graft repair under local anesthesia, classified as Clavien–Dindo grade IIIa. These two patients accounted for a 30-day major complication rate of 22.2% (2/9). The covered stent-graft repair was also recorded as an early reintervention. No spinal cord ischemia, mesenteric ischemia, myocardial infarction, respiratory failure, or stroke occurred within 30 days. The median postoperative length of hospital stay was 14 days (IQR, 12–18; range, 8–49). No unplanned readmissions occurred within 30 days after discharge. Technical success was achieved in all 9 patients (Table 3).

### 3.5. Follow-Up Outcomes

All nine patients adhered to the prescribed postoperative medication and activity recommendations and attended their scheduled follow-up visits. Median follow-up was 9 months (IQR, 6–12; range, 6–15). Four patients (44.4%) had at least 12 months of follow-up. The last clinical follow-up coincided with the last CTA assessment for each patient. No aortic- or graft-related reinterventions occurred after discharge during the available follow-up. At that assessment, all evaluated celiac (8/8), superior mesenteric (9/9), left renal (9/9), right renal (9/9), and splenic (1/1) artery bypasses were patent. Stent-graft exclusion was maintained, no new endoleak was detected, and all nine patients were alive (Table 3).

Because the abdominal aneurysm was excised during the open phase, abdominal aneurysm sac regression was not assessed. Imaging surveillance instead focused on residual aortic disease, stent-graft sealing, bypass patency, anastomotic abnormalities, and endoleak. No additional reintervention was required after treatment of the early right iliac anastomotic leak (Figure 3b).

Long-term imaging surveillance remains scheduled at 12 months after surgery and annually thereafter. CTA assessment includes the residual aorta, stent-graft position and sealing, aortic and iliac anastomotic integrity, visceral and renal bypass patency and stenosis, and endoleaks, including type II endoleak. Earlier imaging is planned for new symptoms or suspected graft-related complications.

## 4. Discussion

In this single-center case series, we evaluated a modified hybrid repair strategy for patients with thoracoabdominal aortic aneurysms (TAAAs) and inadequate distal landing zones. The defining feature of this approach was reconstruction of the distal aortic segment using a prosthetic graft, which provided a new distal sealing zone for subsequent thoracic stent-graft exclusion while allowing retrograde visceral and renal artery revascularization.

No deaths occurred within 30 days after modified hybrid repair in this cohort. Technical success was achieved in all nine patients. No spinal cord ischemia or mesenteric ischemia was observed and all reconstructed visceral and renal bypasses remained patent during follow-up. Postoperative AKI occurred in six patients according to the KDIGO criteria, including one who received CRRT. One patient required early reintervention for a right iliac anastomotic leak. These findings suggest that this strategy is technically feasible in carefully selected patients with challenging distal aortic anatomy.

The primary anatomical challenge addressed by this technique is the absence of an adequate distal sealing zone. Conventional hybrid repair combines visceral debranching with endovascular exclusion and has been increasingly used for patients who are unsuitable for extensive open repair or total endovascular treatment [20]. However, conventional hybrid strategies generally rely on the native distal abdominal aorta or iliac arteries for stent-graft fixation after visceral reconstruction. When aneurysmal degeneration extends to these segments, visceral artery revascularization alone may not resolve the limitation of inadequate distal fixation [2,21]. In such cases, reconstruction of the distal aortic segment may provide an alternative method to establish a durable sealing zone before endovascular exclusion.

The main difference between this modified strategy and conventional hybrid repair is the reconstruction of the distal sealing segment before endovascular exclusion. Rather than relying on diseased native abdominal aorta or iliac arteries, the procedure creates a prosthetic landing zone through open reconstruction.

In addition to providing distal fixation for the thoracic stent graft, the prosthetic graft serves as a common inflow source for retrograde visceral and renal artery bypasses [11,22]. This configuration allows exclusion of the residual proximal thoracoabdominal aneurysmal segment while maintaining perfusion to critical branch vessels.

Compared with conventional open thoracoabdominal replacement, this strategy transfers treatment of the proximal aortic segment from open reconstruction to endovascular exclusion. The abdominal aneurysm and visceral branches are managed under direct vision, whereas the residual proximal suprarenal or thoracoabdominal segment is excluded with a stent graft. This configuration may reduce the need for thoracic exposure and long-segment thoracic aortic replacement [2,21]. However, the prolonged operative time, substantial blood loss, transfusion requirements, and intensive care unit stay in this series reflect its complexity. Its potential value lies in redistributing the technical burden of repair rather than eliminating procedural risk.

Compared with total endovascular repair, modified hybrid repair reduces dependence on native visceral ostial geometry and customized fenestrated or branched devices [23]. A prosthetic inflow source is created during the open phase, and the visceral and renal arteries are reconstructed onto this graft. For patients with extensive distal aneurysmal involvement and inadequate distal landing zones, creation of a prosthetic sealing segment may represent an alternative strategy when conventional endovascular options are limited [24,25].

The principal modification in this configuration is prosthetic reconstruction of the distal sealing segment while establishing inflow for retrograde visceral and renal revascularization. Compared with hybrid strategies that rely on the native distal aorta or iliac arteries for sealing, this approach may expand treatment options when aneurysmal involvement leaves an inadequate distal landing zone. In patients requiring abdominal aortic replacement, aneurysm excision also directly addresses the abdominal component and avoids retaining that portion of the aneurysm sac. The reconstructed graft provides a controlled landing configuration that may reduce concerns about distal sealing failure in diseased native vessels, although a reduction in stent-graft-related complications has not been demonstrated. This strategy may be particularly relevant to selected younger or middle-aged patients with chronic postdissection thoracoabdominal aneurysms extending into the iliac arteries, especially when a previous descending thoracic stent graft provides a usable proximal landing zone. Its suitability must nevertheless be assessed against established open and total endovascular alternatives. These potential advantages entail additional open reconstruction, which may increase bleeding and physiological stress. The substantial blood loss and prolonged operative time in this series underscore the need for experienced surgical, anesthetic, and intensive care teams, but do not establish greater bleeding than conventional hybrid repair [9,26,27].

Perioperative organ injury remains a major concern. Cerebrospinal fluid drainage was used as a prophylactic strategy against postoperative paraplegia [28], and no spinal cord ischemia occurred in this cohort, but the small sample size precludes conclusions regarding risk reduction. The absence of these complications may reflect patient selection, preserved target-vessel patency, appropriate stent-graft planning, and stable perioperative hemodynamics. KDIGO-defined AKI occurred in six patients, including one who received CRRT. These findings highlight renal morbidity as an important concern in this series. Renal artery reconstruction, blood loss, hemodynamic instability, and renal ischemic time may all contribute to postoperative renal dysfunction [5,29]. No adjunctive renal protection technique was applied in this report. Further refinement of spinal cord and renal protection may be warranted in patients with extensive aortic coverage, previous aortic repair, or limited renal reserve [29].

Anastomotic complications represent another source of risk. One patient developed a right iliac artery anastomotic leak within 30 days and underwent emergency covered stent-graft placement. This event shows that surveillance should extend beyond the thoracoabdominal stent graft and visceral bypasses to include abdominal and iliac anastomoses. Potential late complications include anastomotic stenosis, pseudoaneurysm, graft infection, and branch occlusion. The successful endovascular treatment of this leak also illustrates a practical feature of hybrid management: selected complications of open reconstruction may be treated without extensive reoperation.

Early imaging outcomes were stable. All visceral and renal bypasses remained patent, stent-graft exclusion was maintained, and no new endoleak was detected during follow-up. Because the abdominal aneurysm was removed, sac regression was not an applicable endpoint. Surveillance instead focused on residual aortic disease, stent-graft sealing, bypass patency, anastomotic integrity, and reintervention. These findings support early procedural durability but do not establish long-term performance. Late outcomes will depend on both the open and endovascular components of the repair. Continued computed tomography angiography surveillance is therefore required [30].

This study includes all patients who underwent the modified hybrid procedure at our center in 2025, reducing the possibility of selective reporting within that procedural cohort. It describes the operative reconstruction and early clinical and imaging outcomes. These strengths support a detailed assessment of technical feasibility, although they do not establish comparative effectiveness or long-term durability.

This study has several limitations. First, this was a retrospective single-center case series with a limited number of patients and no comparison group; therefore, comparative effectiveness cannot be determined. Second, the follow-up duration was relatively short, and long-term durability of the prosthetic distal landing zone, including potential graft degeneration, migration, or late endoleak, requires further evaluation [27,31]. In addition, the anatomical heterogeneity of TAAA may limit the generalizability of this technique.

## 5. Conclusions

In selected patients with thoracoabdominal aortic aneurysms and inadequate distal landing zones, modified hybrid repair incorporating prosthetic distal landing zone reconstruction and retrograde visceral and renal artery revascularization was technically feasible and achieved favorable early clinical and imaging outcomes. By creating a prosthetic sealing segment, this approach may expand the applicability of hybrid repair in patients whose distal anatomy is unsuitable for conventional endovascular exclusion. Further studies with larger cohorts and longer follow-up are required to evaluate the long-term durability and define the appropriate indications of this strategy.

## Figures and Tables

**Table 1:** Baseline characteristics of 9 patients with thoracoabdominal aortic aneurysms who underwent modified hybrid repair.

Characteristic	Total (*n* = 9)
Age, years	43 (36.5, 70)
Body mass index, kg/m^2^	24.09 ± 3.89
LVEF, %	63 (59, 68)
Hb, g/L	134 (123, 141)
Albumin, g/L	39.7 (38.5, 40.2)
SCr, μmol/L	75 (63, 80)
eGFR, mL/min/1.73 m^2^	108.0 (93.8, 111.4)
TnT, ng/L	6.0 (4.2, 8.9)
CK-MB, ng/mL	1.14 (0.59, 1.61)
BNP, pg/mL	95 (37, 165)
Sex, *n* (%)	
Female	2 (22.2)
Male	7 (77.8)
Ever smoking, *n* (%)	4 (44.4)
Hypertension, *n* (%)	5 (55.6)
Diabetes, *n* (%)	1 (11.1)
Chronic heart failure, *n* (%)	0 (0.0)
Chronic pulmonary disease, *n* (%)	6 (66.7)
History of myocardial infarction, *n* (%)	0 (0.0)
Peripheral artery disease, *n* (%)	2 (22.2)
Intracranial artery disease, *n* (%)	0 (0.0)
Marfan syndrome, *n* (%)	2 (22.2)
Chronic kidney disease, *n* (%)	0 (0.0)
Atrial fibrillation, *n* (%)	0 (0.0)
Aneurysm type, *n* (%)	
Dissecting TAAA	4 (44.4)
Nondissecting TAAA	5 (55.6)
Crawford classification	
I	0 (0.0)
II	5 (55.6)
III	4 (44.4)
IV	0 (0.0)
V	0 (0.0)

Values are mean ± standard deviation, median (interquartile range), or number (%).

**Table 2:** Anatomical and procedural characteristics of patients undergoing modified hybrid repair.

Characteristic	Value
Surgical staging	
single-stage, *n* (%)	9 (100.0)
The maximum diameter of the aneurysm (mm)	58.0 (57.0–64.5)
Iliac artery aneurysm, *n* (%)	2 (22.2)
Involvement of the LSA, *n* (%)	0 (0.0)
Severe bilateral iliac artery stenosis or occlusion, *n* (%)	0 (0.0)
Celiac artery stenosis grade, *n* (%)	
0	6 (66.7)
1	2 (22.2)
2	1 (11.1)
3	0 (0.0)
4	0 (0.0)
SMA stenosis grade, *n* (%)	
0	9 (100.0)
1	0 (0.0)
2	0 (0.0)
3	0 (0.0)
4	0 (0.0)
LRA stenosis grade, *n* (%)	
0	9 (100.0)
1	0 (0.0)
2	0 (0.0)
3	0 (0.0)
4	0 (0.0)
RRA stenosis grade, *n* (%)	
0	7 (77.8)
1	1 (11.1)
2	1 (11.1)
3	0 (0.0)
4	0 (0.0)
Diameter of proximal landing zone	
Descending thoracic aortic stent graft (mm), *n* = 4	27.5 (24.8, 28.0)
Native descending thoracic aortic (mm), *n* = 5	28.0 (23.5, 31.0)
Celiac artery warm ischemia time (min), *n* = 8	19.0 (16.5, 21.5)
Superior mesenteric artery warm ischemia time (min), *n* = 9	18.0 (12.0, 20.0)
Left renal artery warm ischemia time (min), *n* = 9	23.5 (20.5, 29.0)
Right renal artery warm ischemia time (min), *n* = 9	19.0 (17.5, 27.5)
Splenic artery warm ischemia time, min (*n* = 1)	25.0
Intraoperative blood loss (mL)	2500 (1400, 5500)
Autologous blood transfusion volume (mL)	1350 (975, 2125)
Allogeneic blood transfusion volume (mL)	1150 (375, 3650)
Operation time (min)	724 (657.5, 742)
Length of Stay in ICU (d)	8 (5, 9)

LSA, left subclavian artery; CT, celiac trunk; SMA, superior mesenteric artery; LRA, left renal artery; RRA, right renal artery. Visceral arteries are defined as reconstruction of the celiac trunk or splenic artery, superior mesenteric artery and bilateral renal arteries. Values are mean ± standard deviation, median (interquartile range), or number (%). Preoperative native-vessel stenosis was classified as follows: grade 0, no stenosis; grade 1, mild stenosis (>0% to <50%); grade 2, moderate stenosis (50–70%); grade 3, severe stenosis (>70% to <100%); and grade 4, complete occlusion. Grades were assigned for descriptive anatomical reporting. Warm ischemia time was measured from target-vessel clamping to final unclamping and restoration of bypass perfusion, which are presented as median (interquartile range), except for the single splenic artery reconstruction, for which the individual value is reported.

**Table 3:** Early and follow-up outcomes after modified hybrid repair.

Outcome	Value
Primary outcome	
30-day all-cause mortality, *n* (%)	0 (0.0)
Secondary outcome	
Postoperative length of hospital stay, days, median (IQR)	14 (12–18)
Major complication within 30 days, Clavien–Dindo grade ≥III, *n* (%)	2 (22.2)
Paraplegia within 30 days, *n* (%)	0 (0.0)
Reintervention within 1 year after discharge	0 (0.0)
Unplanned readmission within 30 days after discharge, *n* (%)	0 (0.0)
Technical success at completion, *n* (%)	9 (100.0)
Additional early outcomes within 30 days	
Acute kidney injury according to KDIGO criteria, *n* (%)	6 (66.7)
Stage 1	3 (33.3)
Stage 2	2 (22.2)
Stage 3	1 (11.1)
Continuous renal replacement therapy, *n* (%)	1 (11.1)
Spinal cord ischemia, *n* (%)	0 (0.0)
Mesenteric ischemia, *n* (%)	0 (0.0)
Right iliac anastomotic leak, *n* (%)	1 (11.1)
Reintervention within 30 days, *n* (%)	1 (11.1)
Myocardial infarction, *n* (%)	0 (0.0)
Respiratory failure, *n* (%)	0 (0.0)
Stroke, *n* (%)	0 (0.0)
Follow-up outcomes	
Follow-up duration, months, median (IQR), range	9 (6–12), 6–15
Patients alive at last follow-up, *n* (%)	9 (100.0)
Patent celiac artery bypasses at last follow-up, *n*/N (%)	8/8 (100.0)
Patent superior mesenteric artery bypasses at last follow-up, *n*/N (%)	9/9 (100.0)
Patent left renal artery bypasses at last follow-up, *n*/N (%)	9/9 (100.0)
Patent right renal artery bypasses at last follow-up, *n*/N (%)	9/9 (100.0)
Patent splenic artery bypasses at last follow-up, *n*/N (%)	1/1 (100.0)
New endoleak detected during follow-up, *n* (%)	0 (0.0)
Additional reintervention after treatment of the early iliac leak, *n* (%)	0 (0.0)

Values are presented as number (%), number/number (%), median (interquartile range), or range, as appropriate. IQR, interquartile range. Technical success was assessed at completion angiography. Bypass patency denominators represent the number of reconstructed vessels. AKI was classified according to the KDIGO 2012 [18,19] criteria. Each patient was assigned the highest documented stage. Percentages use the entire cohort of nine patients as the denominator. The patient receiving CRRT was included in the stage 3 category. The right iliac anastomotic leak and the early reintervention occurred in the same patient. Major complications were defined as Clavien–Dindo grade III or higher events occurring within 30 days after the index procedure. Each patient was counted once in the overall major complication rate.

## Data Availability

The dataset generated and analyzed is available from the corresponding author upon reasonable request.

## Abbreviations

The following abbreviations are used in this manuscript:

ACT	Activated clotting time
AKI	Acute kidney injury
BNP	B-type natriuretic peptide
CK-MB	Creatine kinase MB isoenzyme
CRRT	Continuous renal replacement therapy
CTA	Computed tomography angiography
eGFR	Estimated glomerular filtration rate
Hb	Hemoglobin
ICU	Intensive care unit
IQR	Interquartile range
KDIGO	Kidney Disease: Improving Global Outcomes
LRA	Left renal artery
LSA	Left subclavian artery
LVEF	Left ventricular ejection fraction
RRA	Right renal artery
SCr	Serum creatinine
SMA	Superior mesenteric artery
TAAA	Thoracoabdominal aortic aneurysm
TEVAR	Thoracic endovascular aortic repair
TnT	Troponin T
